# Ammonium hexa­fluorido­phosphate–18-crown-6 (1/1)

**DOI:** 10.1107/S160053681002920X

**Published:** 2010-07-31

**Authors:** De-Hong Wu, Qi-Qi Wu

**Affiliations:** a College of Chemistry and Chemical Engineering, Southeast University, Nanjing 210096, People’s Republic of China

## Abstract

In the crystal structure of the title compound, NH_4_^+^·PF_6_^−^·C_12_H_24_O_6_, the cation is situated in the 18-crown-6 ring, forming a supra­molecular rotator-stator-like structure held by N—H⋯O hydrogen bonds. The six O atoms of the crown ether lie approximately in a plane [mean deviation 0.2129 (3) Å]; the N atom is displaced by 0.864 (3)Å from the centroid of the 18-crown-6 ring. The slightly distorted tetra­hedral cations further inter­act with the slightly distorted octa­hedral anions *via* inter­molecular N—H⋯F hydrogen bonds.

## Related literature

For background to 18-crown-6 compounds, see: Bajaj & Poonia (1988 ▶); Fender *et al.* (2002 ▶); Kryatova *et al.* (2004 ▶). For related structures. see: Dapporto *et al.* (1996 ▶); Pears *et al.* (1988 ▶).
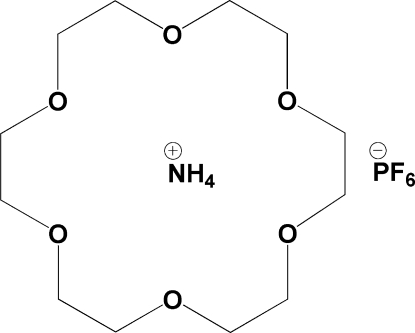


## Experimental

### 

#### Crystal data

NH_4_^+^·PF_6_^−^·C_12_H_24_O_6_
*M* *_r_* = 427.32
Monoclinic, 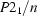

*a* = 12.559 (3) Å
*b* = 8.7352 (17) Å
*c* = 18.6511 (17) Å
β = 94.097 (10)°
*V* = 2040.9 (7) Å^3^
*Z* = 4
Mo *K*α radiation
μ = 0.21 mm^−1^
*T* = 298 K
0.40 × 0.36 × 0.30 mm

#### Data collection

Rigaku Mercury2 diffractometer
Absorption correction: multi-scan (*CrystalClear*; Rigaku, 2005 ▶) *T*_min_ = 0.920, *T*_max_ = 0.940
18388 measured reflections
4014 independent reflections
2740 reflections with *I* > 2σ(*I*)
*R*_int_ = 0.044

#### Refinement

*R*[*F* ^2^ > 2σ(*F* ^2^)] = 0.060
*wR*(*F* ^2^) = 0.160
*S* = 1.04
4014 reflections
239 parameters
H-atom parameters constrained
Δρ_max_ = 0.35 e Å^−3^
Δρ_min_ = −0.27 e Å^−3^

### 

Data collection: *CrystalClear* (Rigaku, 2005 ▶); cell refinement: *CrystalClear*; data reduction: *CrystalClear*; program(s) used to solve structure: *SHELXS97* (Sheldrick, 2008 ▶); program(s) used to refine structure: *SHELXL97* (Sheldrick, 2008 ▶); molecular graphics: *SHELXTL* (Sheldrick, 2008 ▶); software used to prepare material for publication: *SHELXTL*.

## Supplementary Material

Crystal structure: contains datablocks I, global. DOI: 10.1107/S160053681002920X/jh2185sup1.cif

Structure factors: contains datablocks I. DOI: 10.1107/S160053681002920X/jh2185Isup2.hkl

Additional supplementary materials:  crystallographic information; 3D view; checkCIF report

## Figures and Tables

**Table 1:** Hydrogen-bond geometry (Å, °)

*D*—H⋯*A*	*D*—H	H⋯*A*	*D*⋯*A*	*D*—H⋯*A*
N1—H1*D*⋯O1	0.83	2.58	2.993 (3)	113
N1—H1*F*⋯O2	0.78	2.15	2.878 (3)	155
N1—H1*F*⋯O3	0.78	2.43	2.990 (3)	129
N1—H1*C*⋯O4	0.77	2.12	2.876 (3)	169
N1—H1*C*⋯O5	0.77	2.58	3.018 (3)	117
N1—H1*D*⋯O6	0.83	2.05	2.871 (3)	172
N1—H1*E*⋯F1^i^	0.78	2.47	3.179 (4)	151
N1—H1*E*⋯F3^i^	0.78	2.37	3.080 (3)	152

Symmetry code: (i) 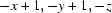.

**Fig. 1.**
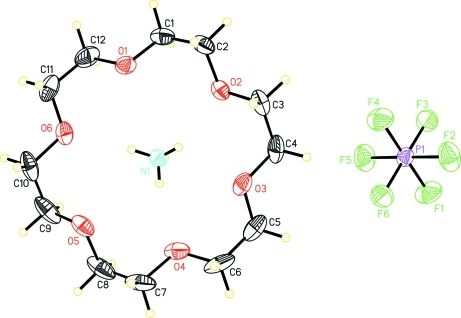
The molecular structure of the title compound, showing the atomic numbering scheme with 30% probability displacement ellipsoids.

**Fig. 2.**
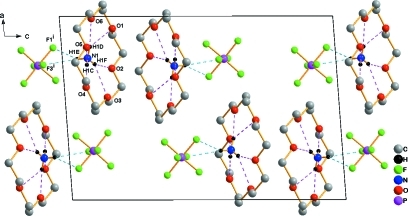
The packing of the title compound viewed along the b axis. The i suffix for atoms F1 and F3 denotes a transformation of (1 - x, 1 - y, -z). Hydrogen atoms not involved in hydrogen bonds (dashed lines) are omitted for clarity.

## supplementary crystallographic information

### Comment

Recently much attention has been devoted to crown ethers due to their ability to form non-covalent, H-bonding complexes with ammonium cations both in solid and in solution (Bajaj *et al.*, 1988; Fender *et al.*, 2002; Kryatova *et al.*, 2004). Both the nature of the ammonium cation (NH_4_^+^,RNH_3_^+^, *R*_2_NH_2_^+^, *etc*.) and the size of the crown ether can work on the stability and stoichiometry of these host–guest complexes. The host molecules combine with the guest species by intermolecular interaction, 18-Crown-6 has a high affinity for RNH_3_^+^cations and most studies of 18-crown-6 and its derivatives invariably showed a 1:1 stoichiometry with RNH_3_^+^ cations. For similar structures, see: Dapporto *et al.*, 1996; Pears *et al.*, 1988. In our laboratory, the title compound has been synthesized and its crystal structure is herein reported.

The title compound crystallizes in the *P*2_1_/n space group with an asymmetric unit consisting of a cationic [(NH_4_)(18-crown-6)]^+^ moiety and an isolated anionic PF_6_^-^(Fig 1). The NH_4_^+^ nests in the 18-crown-6 ring to form a superamolecular rotator-stator-like structure by intramolecular N—H···O hydrogen-bonded interactions between the NH_4_^+^ (H1C, H1D and H1F)and the six oxygen atoms of the crown ether (Fig 2). Intramolecular N—H···O hydrogen distances fall within the normal range: 2.871 (3) and 3.018 (3) Å (Table 1). The six oxygen atoms of the crown ether lie approximately in a plane with the mean deviation of 0.2129 Å, the N atom aparts from the center of the crown ring about 0.864 Å. The slightly distorted tetrahedralcations NH_4_^+^ further interact with F1^i^ and F3^i^ (symmetry code, i: 1 - *x*, 1 - *y*, -*z*) of the slightly distorted octahedral anions PF_6_^-^ (The P—F bond lengths are within the range of 1.51 - 1.60 Å, the F—P—F bond angles are within the range of 87.78 - 92.48°) by intermolecular N—H···F hydrogen bonds (Table 1 and Fig 2).

### Experimental

In room temperature 18-crown-6 (4 mmol, 1.05 g) were dissolved in 50 ml me thanol, after addition of excess hexafluorophosphoric acid to afford a white microcrystallic precipitation for H_3_O^+^?PF_6_^-^(18-crown-6) (about 95% yield). Then dissolve the precipitation again in 50 ml water and addition of excess concentrated ammonia to afford the solution without any participation under stirring at the ambient temperature. Block colorless single crystals suitable for X-ray structure analysis were obtained by the slow evaporation of the above solution after a week in air.

The dielectric constant of the compound as a function of temperature indicates that the permittivity is basically temperature-independent (ε = C/(T–T_0_)), suggesting that this compound is not ferroelectric or there may be no distinct phase transition occurring within the measured temperature range between 93 K and433 K (below the compound melting point 463 K).

### Refinement

H atoms in the crown ring were placed in calculated positions with C—H = 0.97 Å for C*sp^3^* atoms, assigned fixed *U*_iso_ values [*U*_iso_(H) = 1.2*U*eq(C*sp^3^*)] and allowed to ride. The four H atoms of NH_4_^+^ were found with N—H bond distances of between 0.7721 and 0.8282 Å in the difference electron density map.

### Crystal data

**Table:**

NH_4_^+^·PF_6_^−^·C_12_H_24_O_6_	*F*(000) = 896
*M**_r_* = 427.32	*D*_x_ = 1.391 Mg m^−^^3^
Monoclinic, *P*2_1_/*n*	Mo *K*α radiation, λ = 0.71073 Å
Hall symbol: -P 2yn	Cell parameters from 14970 reflections
*a* = 12.559 (3) Å	θ = 3.0–27.8°
*b* = 8.7352 (17) Å	µ = 0.21 mm^−^^1^
*c* = 18.6511 (17) Å	*T* = 298 K
β = 94.097 (10)°	Block, colorless
*V* = 2040.9 (7) Å^3^	0.40 × 0.36 × 0.30 mm
*Z* = 4	

### Data collection

**Table:**

Rigaku Mercury2 diffractometer	4014 independent reflections
Radiation source: fine-focus sealed tube	2740 reflections with *I* > 2σ(*I*)
graphite	*R*_int_ = 0.044
Detector resolution: 13.6612 pixels mm^-1^	θ_max_ = 26.0°, θ_min_ = 3.0°
CCD_Profile_fitting scans	*h* = −15→15
Absorption correction: multi-scan (*CrystalClear*; Rigaku, 2005)	*k* = −10→10
*T*_min_ = 0.920, *T*_max_ = 0.940	*l* = −23→23
18388 measured reflections	

### Refinement

**Table:**

Refinement on *F*^2^	Primary atom site location: structure-invariant direct methods
Least-squares matrix: full	Secondary atom site location: difference Fourier map
*R*[*F*^2^ > 2σ(*F*^2^)] = 0.060	Hydrogen site location: inferred from neighbouring sites
*wR*(*F*^2^) = 0.160	H-atom parameters constrained
*S* = 1.04	*w* = 1/[σ^2^(*F*_o_^2^) + (0.0668*P*)^2^ + 1.0944*P*] where *P* = (*F*_o_^2^ + 2*F*_c_^2^)/3
4014 reflections	(Δ/σ)_max_ < 0.001
239 parameters	Δρ_max_ = 0.35 e Å^−^^3^
0 restraints	Δρ_min_ = −0.27 e Å^−^^3^

### Special details

**Table:**

Geometry. All e.s.d.'s (except the e.s.d. in the dihedral angle between two l.s. planes) are estimated using the full covariance matrix. The cell e.s.d.'s are taken into account individually in the estimation of e.s.d.'s in distances, angles and torsion angles; correlations between e.s.d.'s in cell parameters are only used when they are defined by crystal symmetry. An approximate (isotropic) treatment of cell e.s.d.'s is used for estimating e.s.d.'s involving l.s. planes.
Refinement. Refinement of *F*^2^ against ALL reflections. The weighted *R*-factor *wR* and goodness of fit *S* are based on *F*^2^, conventional *R*-factors *R* are based on *F*, with *F* set to zero for negative *F*^2^. The threshold expression of *F*^2^ > σ(*F*^2^) is used only for calculating *R*-factors(gt) *etc*. and is not relevant to the choice of reflections for refinement. *R*-factors based on *F*^2^ are statistically about twice as large as those based on *F*, and *R*- factors based on ALL data will be even larger.

### Fractional atomic coordinates and isotropic or equivalent isotropic displacement parameters (Å^2^)

**Table:**

	*x*	*y*	*z*	*U*_iso_*/*U*_eq_	
C1	0.9000 (3)	0.5093 (4)	0.18932 (18)	0.0655 (9)	
H1A	0.9538	0.4465	0.2150	0.079*	
H1B	0.8914	0.4736	0.1400	0.079*	
C2	0.7979 (3)	0.4959 (3)	0.22323 (17)	0.0642 (9)	
H2A	0.7804	0.3888	0.2295	0.077*	
H2B	0.8038	0.5441	0.2702	0.077*	
C3	0.6165 (3)	0.5644 (4)	0.20892 (18)	0.0660 (9)	
H3A	0.6212	0.6175	0.2547	0.079*	
H3B	0.5959	0.4592	0.2171	0.079*	
C4	0.5360 (3)	0.6394 (4)	0.1589 (2)	0.0739 (10)	
H4A	0.5371	0.5935	0.1116	0.089*	
H4B	0.4654	0.6249	0.1757	0.089*	
C5	0.4855 (3)	0.8783 (5)	0.1069 (2)	0.0886 (12)	
H5A	0.4135	0.8634	0.1212	0.106*	
H5B	0.4889	0.8384	0.0586	0.106*	
C6	0.5118 (3)	1.0437 (5)	0.1080 (2)	0.0931 (14)	
H6A	0.4575	1.0999	0.0793	0.112*	
H6B	0.5138	1.0819	0.1569	0.112*	
C7	0.6458 (4)	1.2199 (4)	0.0808 (2)	0.0938 (15)	
H7A	0.6540	1.2563	0.1300	0.113*	
H7B	0.5920	1.2822	0.0547	0.113*	
C8	0.7494 (5)	1.2330 (4)	0.0468 (2)	0.0967 (16)	
H8A	0.7437	1.1844	−0.0001	0.116*	
H8B	0.7670	1.3400	0.0403	0.116*	
C9	0.9326 (4)	1.1636 (4)	0.0621 (2)	0.0895 (14)	
H9A	0.9540	1.2689	0.0549	0.107*	
H9B	0.9277	1.1127	0.0158	0.107*	
C10	1.0131 (3)	1.0861 (4)	0.1110 (2)	0.0822 (12)	
H10A	1.0832	1.0972	0.0928	0.099*	
H10B	1.0150	1.1331	0.1582	0.099*	
C11	1.0631 (3)	0.8462 (5)	0.1619 (2)	0.0795 (11)	
H11A	1.0637	0.8851	0.2106	0.095*	
H11B	1.1341	0.8587	0.1453	0.095*	
C12	1.0335 (3)	0.6825 (5)	0.1605 (2)	0.0787 (11)	
H12A	1.0287	0.6452	0.1114	0.094*	
H12B	1.0876	0.6234	0.1880	0.094*	
F1	0.17131 (17)	0.4032 (3)	0.03130 (14)	0.1043 (8)	
F2	0.18504 (19)	0.3563 (3)	0.14865 (13)	0.1016 (8)	
F3	0.26429 (15)	0.2017 (2)	0.07209 (11)	0.0776 (6)	
F4	0.36548 (17)	0.3544 (2)	0.14682 (12)	0.0929 (7)	
F5	0.34845 (19)	0.4032 (3)	0.02988 (13)	0.1027 (8)	
F6	0.27021 (16)	0.5594 (2)	0.10520 (12)	0.0840 (6)	
N1	0.7710 (2)	0.8261 (3)	0.09254 (14)	0.0488 (6)	
H1C	0.7357	0.8983	0.0882	0.13 (2)*	
H1E	0.7676	0.7882	0.0543	0.096 (15)*	
H1D	0.8331	0.8534	0.1039	0.113 (16)*	
H1F	0.7393	0.7719	0.1167	0.14 (2)*	
O1	0.93305 (15)	0.6644 (2)	0.19077 (11)	0.0558 (5)	
O2	0.71616 (16)	0.5684 (2)	0.17870 (10)	0.0539 (5)	
O3	0.55889 (15)	0.7988 (3)	0.15465 (11)	0.0620 (6)	
O4	0.6137 (2)	1.0656 (2)	0.07955 (12)	0.0709 (7)	
O5	0.8311 (2)	1.1606 (2)	0.09173 (11)	0.0653 (6)	
O6	0.98772 (17)	0.9291 (2)	0.11637 (11)	0.0631 (6)	
P1	0.26791 (6)	0.38092 (9)	0.09059 (4)	0.0537 (3)	

### Atomic displacement parameters (Å^2^)

**Table:**

	*U*^11^	*U*^22^	*U*^33^	*U*^12^	*U*^13^	*U*^23^
C1	0.080 (2)	0.0483 (18)	0.068 (2)	0.0199 (16)	−0.0022 (18)	0.0009 (15)
C2	0.094 (3)	0.0443 (17)	0.0532 (18)	−0.0058 (17)	−0.0063 (17)	0.0113 (14)
C3	0.076 (2)	0.0564 (19)	0.068 (2)	−0.0250 (17)	0.0198 (18)	−0.0071 (16)
C4	0.056 (2)	0.080 (2)	0.087 (2)	−0.0299 (18)	0.0119 (18)	−0.030 (2)
C5	0.0465 (19)	0.129 (4)	0.087 (3)	0.008 (2)	−0.0148 (18)	−0.012 (3)
C6	0.073 (3)	0.110 (3)	0.092 (3)	0.054 (2)	−0.021 (2)	−0.008 (2)
C7	0.135 (4)	0.049 (2)	0.089 (3)	0.032 (2)	−0.054 (3)	−0.0103 (19)
C8	0.179 (5)	0.045 (2)	0.060 (2)	−0.015 (3)	−0.035 (3)	0.0138 (17)
C9	0.156 (4)	0.049 (2)	0.070 (2)	−0.025 (2)	0.055 (3)	−0.0036 (17)
C10	0.084 (3)	0.070 (2)	0.099 (3)	−0.039 (2)	0.052 (2)	−0.025 (2)
C11	0.0392 (17)	0.115 (3)	0.086 (3)	−0.0054 (19)	0.0125 (17)	−0.009 (2)
C12	0.054 (2)	0.096 (3)	0.088 (3)	0.029 (2)	0.0155 (18)	0.001 (2)
F1	0.0796 (15)	0.1011 (18)	0.1247 (19)	0.0091 (12)	−0.0443 (14)	−0.0083 (14)
F2	0.1082 (17)	0.0858 (15)	0.1183 (18)	−0.0139 (13)	0.0598 (15)	−0.0182 (13)
F3	0.0747 (13)	0.0560 (12)	0.1020 (15)	−0.0021 (9)	0.0051 (11)	−0.0175 (10)
F4	0.0879 (15)	0.0858 (15)	0.0988 (16)	0.0013 (12)	−0.0361 (12)	−0.0043 (12)
F5	0.0939 (16)	0.1138 (19)	0.1056 (17)	−0.0110 (14)	0.0442 (14)	0.0028 (14)
F6	0.0805 (14)	0.0547 (11)	0.1155 (17)	−0.0061 (10)	−0.0009 (12)	−0.0113 (11)
N1	0.0531 (16)	0.0445 (13)	0.0488 (15)	−0.0016 (13)	0.0033 (11)	−0.0026 (12)
O1	0.0524 (12)	0.0529 (12)	0.0626 (13)	0.0132 (9)	0.0082 (10)	0.0004 (10)
O2	0.0625 (13)	0.0496 (11)	0.0503 (11)	−0.0075 (10)	0.0083 (10)	0.0056 (9)
O3	0.0420 (11)	0.0733 (15)	0.0690 (14)	−0.0032 (10)	−0.0083 (10)	−0.0175 (11)
O4	0.0833 (17)	0.0540 (13)	0.0717 (15)	0.0239 (12)	−0.0210 (13)	−0.0119 (11)
O5	0.1022 (18)	0.0461 (12)	0.0478 (12)	−0.0099 (12)	0.0068 (12)	0.0064 (9)
O6	0.0569 (13)	0.0651 (13)	0.0689 (14)	−0.0166 (11)	0.0165 (11)	−0.0052 (11)
P1	0.0441 (4)	0.0524 (5)	0.0643 (5)	−0.0032 (4)	0.0011 (3)	−0.0086 (4)

### Geometric parameters (Å, °)

**Table:**

C1—O1	1.416 (4)	C8—H8A	0.9700
C1—C2	1.475 (5)	C8—H8B	0.9700
C1—H1A	0.9700	C9—O5	1.424 (5)
C1—H1B	0.9700	C9—C10	1.477 (6)
C2—O2	1.422 (4)	C9—H9A	0.9700
C2—H2A	0.9700	C9—H9B	0.9700
C2—H2B	0.9700	C10—O6	1.413 (4)
C3—O2	1.410 (4)	C10—H10A	0.9700
C3—C4	1.478 (5)	C10—H10B	0.9700
C3—H3A	0.9700	C11—O6	1.423 (4)
C3—H3B	0.9700	C11—C12	1.478 (5)
C4—O3	1.425 (4)	C11—H11A	0.9700
C4—H4A	0.9700	C11—H11B	0.9700
C4—H4B	0.9700	C12—O1	1.426 (4)
C5—O3	1.417 (4)	C12—H12A	0.9700
C5—C6	1.482 (6)	C12—H12B	0.9700
C5—H5A	0.9700	F1—P1	1.594 (2)
C5—H5B	0.9700	F2—P1	1.569 (2)
C6—O4	1.432 (5)	F3—P1	1.6029 (19)
C6—H6A	0.9700	F4—P1	1.572 (2)
C6—H6B	0.9700	F5—P1	1.583 (2)
C7—O4	1.407 (4)	F6—P1	1.583 (2)
C7—C8	1.492 (6)	N1—H1C	0.7721
C7—H7A	0.9700	N1—H1E	0.7840
C7—H7B	0.9700	N1—H1D	0.8282
C8—O5	1.427 (5)	N1—H1F	0.7827
			
O1—C1—C2	109.3 (2)	C10—C9—H9A	109.6
O1—C1—H1A	109.8	O5—C9—H9B	109.6
C2—C1—H1A	109.8	C10—C9—H9B	109.6
O1—C1—H1B	109.8	H9A—C9—H9B	108.1
C2—C1—H1B	109.8	O6—C10—C9	109.9 (3)
H1A—C1—H1B	108.3	O6—C10—H10A	109.7
O2—C2—C1	109.1 (2)	C9—C10—H10A	109.7
O2—C2—H2A	109.9	O6—C10—H10B	109.7
C1—C2—H2A	109.9	C9—C10—H10B	109.7
O2—C2—H2B	109.9	H10A—C10—H10B	108.2
C1—C2—H2B	109.9	O6—C11—C12	109.0 (3)
H2A—C2—H2B	108.3	O6—C11—H11A	109.9
O2—C3—C4	108.9 (3)	C12—C11—H11A	109.9
O2—C3—H3A	109.9	O6—C11—H11B	109.9
C4—C3—H3A	109.9	C12—C11—H11B	109.9
O2—C3—H3B	109.9	H11A—C11—H11B	108.3
C4—C3—H3B	109.9	O1—C12—C11	109.2 (3)
H3A—C3—H3B	108.3	O1—C12—H12A	109.8
O3—C4—C3	109.7 (2)	C11—C12—H12A	109.8
O3—C4—H4A	109.7	O1—C12—H12B	109.8
C3—C4—H4A	109.7	C11—C12—H12B	109.8
O3—C4—H4B	109.7	H12A—C12—H12B	108.3
C3—C4—H4B	109.7	H1C—N1—H1E	104.9
H4A—C4—H4B	108.2	H1C—N1—H1D	108.4
O3—C5—C6	109.5 (3)	H1E—N1—H1D	110.2
O3—C5—H5A	109.8	H1C—N1—H1F	104.0
C6—C5—H5A	109.8	H1E—N1—H1F	105.7
O3—C5—H5B	109.8	H1D—N1—H1F	122.3
C6—C5—H5B	109.8	C1—O1—C12	111.4 (2)
H5A—C5—H5B	108.2	C3—O2—C2	112.3 (2)
O4—C6—C5	109.3 (3)	C5—O3—C4	112.9 (3)
O4—C6—H6A	109.8	C7—O4—C6	112.6 (3)
C5—C6—H6A	109.8	C9—O5—C8	112.9 (3)
O4—C6—H6B	109.8	C10—O6—C11	113.1 (3)
C5—C6—H6B	109.8	F2—P1—F4	92.48 (14)
H6A—C6—H6B	108.3	F2—P1—F6	91.19 (12)
O4—C7—C8	108.9 (3)	F4—P1—F6	91.52 (12)
O4—C7—H7A	109.9	F2—P1—F5	177.92 (14)
C8—C7—H7A	109.9	F4—P1—F5	89.31 (14)
O4—C7—H7B	109.9	F6—P1—F5	89.83 (13)
C8—C7—H7B	109.9	F2—P1—F1	89.19 (15)
H7A—C7—H7B	108.3	F4—P1—F1	177.60 (14)
O5—C8—C7	109.2 (3)	F6—P1—F1	90.16 (12)
O5—C8—H8A	109.8	F5—P1—F1	88.99 (15)
C7—C8—H8A	109.8	F2—P1—F3	90.29 (11)
O5—C8—H8B	109.8	F4—P1—F3	90.49 (11)
C7—C8—H8B	109.8	F6—P1—F3	177.45 (13)
H8A—C8—H8B	108.3	F5—P1—F3	88.63 (12)
O5—C9—C10	110.1 (3)	F1—P1—F3	87.78 (12)
O5—C9—H9A	109.6		

### Hydrogen-bond geometry (Å, °)

**Table:**

*D*—H···*A*	*D*—H	H···*A*	*D*···*A*	*D*—H···*A*
N1—H1D···O1	0.83	2.58	2.993 (3)	113
N1—H1F···O2	0.78	2.15	2.878 (3)	155
N1—H1F···O3	0.78	2.43	2.990 (3)	129
N1—H1C···O4	0.77	2.12	2.876 (3)	169
N1—H1C···O5	0.77	2.58	3.018 (3)	117
N1—H1D···O6	0.83	2.05	2.871 (3)	172
N1—H1E···F1^i^	0.78	2.47	3.179 (4)	151
N1—H1E···F3^i^	0.78	2.37	3.080 (3)	152

Symmetry codes: (i) −*x*+1, −*y*+1, −*z*.
